# Supplementary material for: Empirical methods for controlling false positives and estimating confidence in ChIP-Seq peaks
Source: BMC Bioinformatics. 2008 Dec 5;9:523. doi: 10.1186/1471-2105-9-523 (PMC2628906; doi:10.1186/1471-2105-9-523)
Supplement: Additional File 1 — A variety of html documents from the USeq web site detailing the available applications, their best usage, output file type descriptions, command line menus, etc. [file 1471-2105-9-523-S1.zip › USeqUserGuides/JavaDoc.zip!/JavaDoc/trans/cel/CelMapper.html]

CelMapper


|  |  |  |
| --- | --- | --- |
| **Overview** \| **Package** \| **Class** \| **Use** \| **Tree** \| **Deprecated** \| **Index** \| **Help** | |  |
| **PREV CLASS**   **NEXT CLASS** | **FRAMES**    **NO FRAMES**     **All Classes** |  |
| SUMMARY: NESTED \| FIELD \| CONSTR \| METHOD | DETAIL: FIELD \| CONSTR \| METHOD |  |


---


## trans.cel Class CelMapper

```
java.lang.Object
  trans.cel.CelMapper
```

---

``` public class CelMapper extends java.lang.Object ```

Saves float[]s of intensity values for each cel file ordered by the tpmapFeature file.
If useMMData is true, will double array size and set mm value afterward.

---

**Constructor Summary**

`CelMapper(MapFeature[] features,
boolean useMMData,
boolean printStats)`


| **Method Summary** | |
| --- | --- |
| `int` | `getSizeMappedFeatures()` |
| `java.io.File[]` | `mapCelFiles(java.io.File[] files)` |
| `boolean` | `mapCelFiles(java.io.File[] files, java.io.File[] matchedSaveAsFiles)`             Maps float[][] to MapFeature[][] saving under the index matched File in matchedSaveAsFiles as a MultiSetQuantile[]. |

**Methods inherited from class java.lang.Object**

`equals, getClass, hashCode, notify, notifyAll, toString, wait, wait, wait`

**Constructor Detail**

### CelMapper

```
public CelMapper(MapFeature[] features,
                 boolean useMMData,
                 boolean printStats)
```


**Method Detail**

### mapCelFiles

```
public java.io.File[] mapCelFiles(java.io.File[] files)
```

---


### mapCelFiles

```
public boolean mapCelFiles(java.io.File[] files,
                           java.io.File[] matchedSaveAsFiles)
```

:   Maps float[][] to MapFeature[][] saving under the index matched File in matchedSaveAsFiles as a MultiSetQuantile[].
    Will perform a max(PM-MM,1) transform if useMMData is true.

---


### getSizeMappedFeatures

```
public int getSizeMappedFeatures()
```


---


|  |  |  |
| --- | --- | --- |
| **Overview** \| **Package** \| **Class** \| **Use** \| **Tree** \| **Deprecated** \| **Index** \| **Help** | |  |
| **PREV CLASS**   **NEXT CLASS** | **FRAMES**    **NO FRAMES**     **All Classes** |  |
| SUMMARY: NESTED \| FIELD \| CONSTR \| METHOD | DETAIL: FIELD \| CONSTR \| METHOD |  |


---
